# Abundant Small Protein ICARUS Inside the Cell Wall of Stress-Resistant Ascospores of *Talaromyces macrosporus* Suggests a Novel Mechanism of Constitutive Dormancy

**DOI:** 10.3390/jof7030216

**Published:** 2021-03-17

**Authors:** Jan Dijksterhuis, Timon Wyatt, Micha Hanssen, Elena Golovina, Folkert Hoekstra, Luis Lugones

**Affiliations:** 1 Westerdijk Fungal Diversity Institute, Uppsalalaan 8, 3584 CT Utrecht, The Netherlands; timonwyatt@gmail.com (T.W.); Micha.Hanssen@gmx.net (M.H.); 2 Laboratory of Biophysics, Wageningen University and Research, 6708 PB Wageningen, The Netherlands; elenagolovina@gmail.com (E.G.); folkertahoekstra@gmail.com (F.H.); 3 Microbiology, Department of Biology, Utrecht University, Padualaan 8, 3584 CH Utrecht, The Netherlands; l.g.lugones@uu.nl

**Keywords:** heat-resistant fungi, ascospore, dormancy, food spoilage, stress resistance, germination, *Talaromyces*

## Abstract

Ascospores of *Talaromyces.macrosporus* belong to the most stress resistant eukaryotic cells and show a constitutive dormancy, i.e., no germination occurs in the presence of rich growth medium. Only an extreme trigger as very high temperature or pressure is able to evoke synchronized germination. In this study, several changes within the thick cell wall of these cells are observed after a heat treatment: (i.) a change in its structure as shown with EPR and X-ray diffraction; (ii.) a release of an abundant protein into the supernatant, which is proportional to the extent of heat activation; (iii.) a change in the permeability of the cell wall as judged by fluorescence studies in which staining of the interior of the cell wall correlates with germination of individual ascospores. The gene encoding the protein, dubbed ICARUS, was studied in detail and was expressed under growth conditions that showed intense ascomata (fruit body) and ascospore formation. It encodes a small 7–14 kD protein. Blast search exhibits that different *Talaromyces* species show a similar sequence, indicating that the protein also occurs in other species of the genus. Deletion strains show delayed ascomata formation, release of pigments into the growth medium, higher permeability of the cell wall and a markedly shorter heat activation needed for activation. Further, wild type ascospores are more heat-resistant. All these observations suggest that the protein plays a role in dormancy and is related to the structure and permeability of the ascospore cell wall. However, more research on this topic is needed to study constitutive dormancy in other fungal species that form stress-resistant ascospores.

## 1. Introduction

Heat-resistant fungi cause spoilage of food products after heat treatments, such as pasteurization. This is the result of the presence of extreme stress resistant ascospores [1,2] that are formed in a homothallic (i.e., self-fertile) fashion, as in the case of the model systems *Talaromyces macrosporus* and *Aspergillus fischeri* (earlier described as *Neosartorya fischeri*) [3,4] or in a heterothallic fashion (as is described forthe fungi *Paecilomyces variotii* and *Aspergillus fumigatus* [5,6]). These ascospores are characterized by a dormant state that is independent of the presence of nutrients (as e.g., [7,8]). This constitutive dormancy (as defined by [9]) can be broken by extreme triggers as high temperature (85 °C, e.g., [10]) or high pressure (6000 Bar, [11,12]). Spoilage of food products after pasteurization is a consistent problem in food industry [13,14,15].

*T. macrosporus* is a model system for heat-resistant ascospores. Ascospores are formed inside numerous asci in ascomata, present in large numbers on the growth medium after 40 days of cultivation. Homogenous suspensions with high numbers of spores are easily obtained and can be studied with respect to their properties. Many studies on D-values of ascospores of different species of heat-resistant fungi have been performed in the past [2]. A D_T_-value expresses the time needed, at temperature T, to heat-inactivate 90% of the spores. A comparison of nearly all the data compiled from these studies indicate that ascospores originating from different species exhibit varying heat resistance. *T. macrosporus* belongs to the most heat-resistant fungi [2].

Ascospores of *T. macrosporus* are characterized by an extremely thick cell wall and dense cytoplasm containing highly accumulated trehalose [3,4,16,17]. Germination of ascospores is activated and synchronized bya heat treatment at 80–85 °C for 5–10 min, temperatures that are characteristic for a pasteurization treatment. Kikoku [18,19] showed that activation became more pronounced when the temperature increased between 81 and 91 °C. Upon heat activation, germinating ascospores show a very fast degradation of trehalose, accompanied by a decrease in intracellular (micro)viscosity as measured by means of electron spin resonance [3,4,17]. These observations suggest that cytoplasmic viscosity parameters play an important role in prolonged dormancy and that degradation of compatible solutes is an important stage in the renormalization of the ascospore towards a growing fungal vegetative cell. Subsequently, ascospores of *T. macrosporus* show a fast shedding of the external thick cell wall, increase of respiration and subsequent swelling of the spore [17,20]. Remarkably, shedding of the outer cell wall, could be blocked by the respiration blocker sodium azide. To complicate things more, after washing away azide, trehalose breakdown had occurred and ESR measurements still suggested high micro(viscosity) and low respiration.After shedding, microviscosity dropped and respiration increased strongly.

The breaking of dormancy in heat-resistant fungi is not addressed in great detail. Ascospores of *T. macrosporus* do not measurably consume any oxygen [16] and have more profound blockage of metabolic activity compared to conidia (as is observed in the case of *Aspergillus niger* that shows some respiration [16,21]). When ascospores are activated by heat and subsequently freeze-dried or frozen (either −20 °C or −196 °C), ascospores remain activated upon rewetting and cultivation [22]. It is of interest to realize that stress resistance of ascospores remains high only when drying or freezing was applied directly after the heat activation treatment. First, dormancy is broken, then an autonomous process leads to germination characterized by intracellular conditions that conduce high metabolic activity at low concentrations of compatible solutes and low microviscosity. Further investigations into the processes following the heat-activation of these spores may lead to new ways of preventing food spoilage by *T. macrosporus* and other heat-resistant fungi.

However, the nature of (heat) activation of extreme stress-resistant fungal ascospores is never addressed. In this contribution, we provide data that suggest a novel mechanism in which the impermeability of the thick outer layer of the cell wall is conveyed by a small protein, ICARUS, that is present either within or around the spore.

## 2. Materials and Methods

### 2.1. Fungal Strains

Cultures of the fungus *Talaromyces macrosporus* (CBS 130.89 [23]) were grown on oatmeal agar plates for 40 days at 30 °C. The ascomata were scraped off of the plates and suspended in 20 mL of N-(2-Acetamido)-2-aminoethane-sulfonic acid (ACES) buffer (Sigma, pH 6.8, 10 mM) supplemented with 0.05% TWEEN 80. The ascomata and asci were ruptured by either suction through a 10 mL syringe with a 0.9 mm hypodermic needle or short vortexing with a mixture of 1 mm and 0.1 mm glass beads. After the asci had been loosened, the suspension was filtered through a 30 mL syringe filled with 5–10 mL sterile glass wool at the bottom. Then, the suspension was washed 2–4 times with ACES buffer by centrifugation and the pellet was resuspended in fresh ACES buffer. To count the spores a hemocytometer was used.

### 2.2. Preparation of a Cell Wall Fraction

Costar tubes (50 mL) were filled with 1 mm glass beads to about 5 to 10 mL. Subsequently, the spore solution (typically 10^8^ spores/mL) was added as a volume that did not exceed the level of glass beads. The suspension was vortexed for 3 minwith intermittant cooling on ice. By this method an average of 96% of the spores could be broken as was judged by microscopical examination (see Supplementary Figure S1).

### 2.3. ESR and X-ray Analysis

For the ESR sample preparation, 40 day-old cultures were isolated as described above. After the last washing step the spores were suspended in 10 mL ACES buffer. A quantity of 4.5 mL of the spore suspension was activated (7 min at 85 °C). Both heat-activated and non-activated spores were broken as described above. After breaking the spores, they were taken out of the Costar tubes with a 10 mL syringe and distributed over 6 Eppendorf tubes (three for the activated spores and three for the non-activated spores). The pellets were washed 4 times with Milli-Q water (centrifuged for 30 s at 15.000× *g* in an Eppendorf centrifuge) and resuspended in the vial. Two of the six tubes (one activated and one non-activated) were centrifuged and the pellet was washed with Milli-Q. The other four cups were also centrifuged and resuspended in 500 μL of 2% SDS. After that they were incubated for 10 min at 100 °C. Then, they were washed with Milli-Q again for four times. Two of the four pellets were subsequently resuspended in Milli-Q- while the others were resuspended in 500 µL 1 N KOH and incubated for 20 min at 60 °C. Afterwards the two KOH treated solutions were washed again for four times and then resuspended in 500 uL Milli-Q.

For ESR, all samples were prepared with perdeuterated TEMPONE (PDT,-4-oxo-2,2,6,6-tetramethylpiperidine-N-oxyl, 2 mM, obtained from Prof. Igor Grigoriev, Novosibirsk, Russia) and potassium ferricyanide (120 mM) was added and the spectra were recorded. The samples were placed in a glass capillary (i.d. 2.5 mm) flame-sealed from one end and then the capillary with a sample was transferred into the quartz tube that was fixed in the ESR cavity for spectra recording by means of a ESR spectrometer ELEXYS 500 (Bruker EAS GmbH, Hanau, Germany). ESR spectra were recorded at 2 mW microwave power, the modulation amplitude was 0.2–1 gauss depending on the shape of the spectrum.

For X-ray diffraction studies, powder diffraction recordings [24] freeze dried cell wallswre packed into small holes in plastic specimens discs, which were mounted on a X-ray collimator and exposed to nickel-filtered copper (CuKa) radiation. The X-ray tube was operated at 38 kV, 23 mA and the exposure time was 30 min. Specimen to film distance was 40 cm.

### 2.4. Protein Release during Heat Activation

A quantity of 375 μL of spore suspension (10^8^ spores/mL) was brought in Eppendorf cups. To activate the spores, the Eppendorf cups were heated at 85 °C for 7 min in a Julabo waterbath (Julabo, GmbH, Seelbach, Germany). Then, the spores were spun down for 20 s at 13.000 rpm. A quantity of 300 μL of denaturing sample buffer was added to the each of the supernatants. The samples were heated to 100 °C for 5 min and run on a 15% SDS-PAGE gel for 50 min at 200 Volts. For N-terminal sequence analysis of the protein, the SDS-PAGE gel was blotted on PVDF membrane for 4.5 min at 60 V. The blotting buffer used was the same as the Electrophoresis buffer, i.e., Tris/glycine/SDS (TGS) but now with 15 mL methanol added. Blots were stained with Coomassie Brilliant Blue R250 in 10% (*v*/*v*) acetic acid, 30% (*v*/*v*) methanol and de-stained in the same solvent. For staining of the SDS-PAGE gel Coomassie Brilliant Blue was used for one hour. For the subsequent destaining 10% methanol, 10% acetic acid was used. The de-staining was done overnight. The staining of the blot was also done with Coomassie Brilliant Blue, for 5 min. Amino-terminal sequencing was carried out on a cut-out band from the gel blot with a pulse liquid sequenator on-line, connected to a phenylthiohydantoin analyser (Applied Biosystems, Foster City, CA, USA).

The 2D gel electrophoresis was conducted according to [25].

### 2.5. Protein Measurement by BCA

Protein levels in supernatant of ascospores suspensions were measured using a BCATM Protein Assay Kit (Pierce, Rockford, IL, USA) and measuring staining of supernatants at 590 nm. Bovine Serum Albumin was used for calibration.

### 2.6. Staining of Ascospores by Carboxy Fluorescein

Ascospores originating from 33-day-old to 49-day-old cultures were stained in 2.9–9.5 mM carboxy fluorescein in ACES bufferfor 15 min and stained at 30 °C and 160 rpm agitation. Ascospores were washed in buffer and fluorescence was assessed using a Zeis Axioskop (Zeiss, Oberkochen, Germany) equipped with Filterblock II (09), 450–490 nm, FT 510, LP 520. Micrographs were taken with the Axiocam software (Zeiss, Oberkochen, Germany). Cells were observed in liquid, but in cases that the correlation between cell wall staining and germination was assessed, stained ascospores were immobilized on a thin layer of agar on an objective glass and investigated by light microscopy after 6 h. Fluorescence intensity was quantified, using the original pictures, as arbitrary units using the Adobe Photoshop software.

### 2.7. Total RNA Isolation

For RNA isolation, mycelium of 20–30-day-old agar cultures of *T. macrosporus* was used, frozen in liquid nitrogen and pulverized using a dismembrator (TissueLyser, QIAGEN Benelux B.V., Venlo, The Netherlands) for two times 45 s at 2000 rpm. Subsequently, the broken mycelium was transferred to a cooled Eppendorf cup and total RNA was extracted with TriZol (Invitrogen, ThermoFischer Scientific, Landsmeer, The Netherlands) following the manufacturer’s protocol.

### 2.8. cDNA Isolation

In order to obtain the sequence of ICARUS its cDNA was isolated through RT-PCR.

RT was done on 2 μg total RNA using AMV Reverse transcriptase (Roche, Merck KGaA, Darmstadt, Germany) following the instructions of the manufacturer. As reverse primer for the cDNA synthesis an oligo dT primer was used (5′ TTA ATT TTT TTT TTT TTT TTT TTT TTV 3′).

For the PCR reaction to generate the second strand, a primer derived from the N-terminal sequence of the protein was used (5′ CCY ACY CTS ACB GAY GAY GCB GAY TAY 3′) together with the reverse primer used for first strand synthesis. The thermal cycling program was 94 °C (4 min); {94 °C (20 s); 45 °C (20 s); 72 °C (2 min)} × 35 times; 72 °C (10 min). The obtained band was cloned in pUC20 and sent for sequencing.

### 2.9. Chromosomal DNA Isolation

Genomic DNA was isolated from *T. macrosporus* ascospores (5 × 10^6^/mL) that had germinated for 16 h at 30 °C in 100 mL malt extract broth and shaken at 200 rpm. To the culture fluid another 100 mL of fresh medium was added to 10 mL of blended culture and inoculated for an additional 36–48 h. The mycelium was spun down and frozen in liquid nitrogen. The mycelium was pulverized in a Tissuelyser. To approximately 3 mL of pulverized mycelium, 5 mL DNA extraction buffer, 80 μL Proteinase K (10 mg/mL) and 100 μL β-mercapto-ethanol were added and incubated for 30 min at 55 °C. After centrifugation (10 min, 10,000× *g*), the supernatant was placed in a clean tube and 1 volume isopropanol was added. The formed pellet (5 min, 10,000× *g*) was washed with 70% ethanol, dried and dissolved in TE (10 mM Tris, 3 mM EDTA, pH 8.0). 100 μg/mL RNAse was added to degrade the RNA (15 min, 37 °C). The amount of DNA was assessed by gel electrophoresis and further purified on a silica column and stored at 4 °C.

### 2.10. DNA and RNA Hybridizations

DNA and RNA hybridizations were conducted according to [26].

### 2.11. PCR Walking with Splinkerette’s

PCR-walking with splinkerettes [27] was used to obtain the flanking regions of the ICARUS gene. Splinkerette bottom and top (Supplementary Table S1) were annealed in a 100 μL annealing mixture in water consisting of 8 μM splinktop primer; 8 μM splinkbottom primer; 10 mM Tris (pH 7.5) and 5 mM MgCl_2_. The mixture was incubated at 94 °C for 4 min and slowly chilled till room temperature.

Digested gDNA of *T. macrosporus* was ligated with a 15 times molar excess of splinkerettes in 20 μL for 4.5 h at room temperature. Then, a primary and a secondary PCR were performed. The first PCR was done on 1μL ligation mixture (30 ng) with primers (Supplementary Table S1) n1 (50 pmol), which annealed on the splinkerette and playipr1 or playipf1 (10 pmol), which annealed on each of the ends of the known sequence. A touchdown cycling protocol was used (30 s, 95 °C and 15 s during each cycle, annealing for 1 min at 71 °C, but decreasing by 2 °C per cycle until 61 °C, 2 min at 72 °C (cycles 1–10), then 4 min (cycles 11–20) and finally, 6 min (cycles 21–30). A second PCR was done using 1 μL primary PCR mixture and nested primers (n2 and playif2 or playir2 depending on which of the two specific primers had worked in the first PCR) using the same parameters as in the first PCR. Amplified DNA was separated on a gel and fragments with the right size were cut out and purified, cloned and sent for sequencing.

### 2.12. Construction of a Deletion Plasmid

The ICARUS gene was disrupted by replacing the gene with an incomplete copy, which missed the promoter and the first part of the coding region, including an encoded signal peptide for secretion (as shown in Supplementary Figures S3 and S5). Primers for amplifying the flanks upstream and downstream of the region to be deleted were designed based on the sequence of the fragment isolated through the splinkerette protocol. Amplified flanks were cloned in vector pUC20. Both fragments were cut out of the plasmids with the proper restrictions enzymes and ligated in the vector Pan7–1 using the same restriction enzymes, which allowed for directional cloning of both fragments. The upstream flank was ligated at the end of the hygromycin resistance cassette using the restriction sites HindIII and XbaI. The downstream flank was ligated 500 bp upstream of hygromycin resistance cassette start, using the restriction sites NheI and BglII.

### 2.13. Protoplast Preparation

Protoplast isolation and transformation were done based on [28]. Mycelium was grown in malt extract medium (MEB) inoculated with 5 × 10^6^ heat-activated ascospores and grown for 2 days (30 °C, 250 rpm). The culture was homogenized for 30 s in a Waring blender at the low stand and the homogenate was used to inoculate fresh malt extract medium. The new culture was further grown at 30 °C for 16 h under shaking (250 rpm). 50 mL culture were transferred to a Falcon tube and spun down at 4500× *g*. The pellet was washed twice with 1 M MgSO_4_ and resuspended in cell wall lysing buffer (Lysing Enzymes, Applied Plant Research, Wageningen University, 10 mg) dissolved in 20 mL 1 M MgSO_4_ and 200 μL 0.5 M malate buffer (pH 5.8) The mixture was incubated at 26 °C on a shaker at 70 rpm for approximately 3 h. The protoplast suspension was then filtered through sterile glass wool to remove the mycelium debris. To the clean protoplast suspension cold STC (1.2 M sorbitol, 10 mM Tris-HCl pH 7.5, 10 mM CaCl_2_) was added to a total volume of 45 mL. The protoplasts were pelleted by centrifugation at 800× *g* at 4 °C in a swing out rotor (20 min) and washed with 45 mL cold STC. The protoplasts were resuspended in STC buffer to a final concentration of 10^8^/mL.

### 2.14. Transformation of T. macrosporus Protoplasts

For each transformation, 200 μL of the protoplast suspension were used. To the protoplasts, 1 μg DNA was added. After the addition of 50 μL PEG buffer (25% PEG-6000, 50 mM CaCl2, 10 mM Tris/HCl pH 7.5) the mixture was gently shaken and incubated at RT (room temperature) for 20 min. After the incubation, 2 mL of PEG buffer was added and gently mixed. The mixture was incubated for another 5 min at RT. Subsequently, 4 mL of STC was added. Finally, selective malt extract-top agar (malt extract, pH 6.0, 0.95 M sucrose, 0.6% Low Melting Point Agarose, Hygromycin 200 μg/μL) was added to a total volume of 15 mL. After mixing the suspension was poured on selective plates (malt extract pH 6.0, 0.95 M sucrose, 1.2% Agar, Hygromycin 200 μg/μL). The plates with protoplasts were incubated upside down at 30 °C for 3 days.

### 2.15. Expression of ICARUS under Several Growth Conditions

Heat-activated ascospores were inoculated on different growth media. RNA was isolated from fungal hyphae and ascomata. Total RNA was ran on denaturing gel, blotted, and hybridized with probes specific for ICARUS by a Northern blotting procedure. As growth media were used: Oatmeal agar, Hay extract, Horse dung medium, Cherry medium and Malt extract medium as described in [29]. For development of structures in the dark, microtiter plates containing the liquid media were wrapped in tin foil and kept within a closed carboard box in order to study the effect of darkness on ascomata formation.

### 2.16. Scanning Electron Microscopy and Cryo-Planing

cryoSEM was conducted according to [2] and cryo-planing was conducted as described in [17]. 

## 3. Results

### 3.1. Heat Activation of T. macrosporus Ascospores Only Occurs above 70 °C

**Figure 1:**
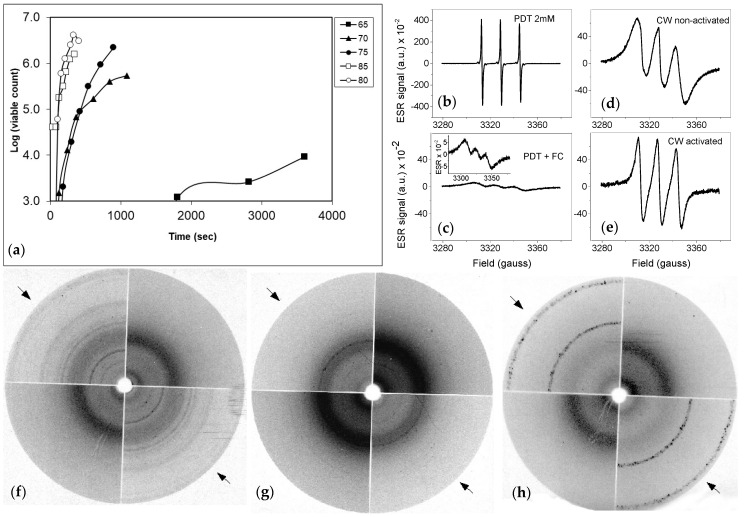
Cell walls of ascospores of *T. macrosporus* change in structure after heat activation. (**a**) Heat-activation of ascospores is only observed at temperatures above 70 °C and increase in speed at higher temperatures; (**b**,**c**) ESR spectra of PDT plus FC added. Note that the scale of PDT signal in liquid is large (**b**), while it is very reduced in the presence of FC (**c**). The inset shows the shape of the signal including three peaks; (**d**,**e**) Spectra of cell walls from dormant and activated spores. The signal of PDT of activated cell walls has more narrow lines, indicating that a structural change has taken place during activation. (**f**–**h**) X-ray diffraction patterns of broken, freeze dried cell walls. (**f**) shows diffraction patterns of broken cell walls of ascospores, (**g**) shows diffraction patterns of broken cell walls after they have been treated with 2% SDS at 100 °C for ten minutes. (**h**) shows the diffraction patterns of spores that have had the same SDS treatment in (**g**) but with an additional treatment with 1 N KOH of 20 min at 60 °C. Arrows indicate two diffraction patterns of activated spores; the other two quarters of the circles represent diffraction patterns of non-activated spores.

Initially, we characterized the threshold temperature required for heat activation of dormant ascospores. Ascospore suspensions in ACES buffer were heat treated and subsequently plated out on agar surfaces, when a temperature of 65 °C was applied, only after a 60 min heat-treatment colony formation of ascospores had increased 10-fold compared to untreated ascospores (Figure 1a). At 70 and 75 °C, a hundred to thousand-fold increase was observed within 20 min of treatment. At 80 and 85 °C, a 5 min treatment resulted in an over 1000-fold increase. This indicates that effective activation of the majority of the ascospores needs temperatures at or above 80 °C. 

### 3.2. Electron Spin Resonance Spectroscopy Reveals Structural Changes after Heat Activation

In order to study if changes in the cell wall structure had occurred after a heat treatment, we studied cell wall preparations of dormant and activated cell walls by means of electron spin resonance spectroscopy (ESR) using the spin probe perdeuterated TEMPONE (PDT). PDT produces a spectrum of three resonance lines in a changing magnetic field (Figure 1b). Addition of potassium ferricyanide (FC) to a solution of PDT in a final concentration of 120 mM, causes considerable broadening of the spectral lines (Figure 1c) due to spin-spin interactions occurring during collisions of PDT molecules with FC ions. Ascospores were broken and the cell walls were spun down and washed with ultra-pure water. Addition of cell wall material from dormant spores caused a reduced effect of FC on broadening of the PDT signal (Figure 1d). This means that the collisions between PDT and FC are partly blocked within the cell wall. The signal obtained in the case of cell walls of activated cells was even less broadened (Figure 1e).

These changes are due to a lesser accessibility of PDT for collisions with the FC ions. This might indicate that structural changes cause the formation of more molecular cavities allowing more freedom of rotation of PDT molecules, but not for the larger FC ions, resulting in motional narrowing of their spectral lines.

### 3.3. X-ray Diffraction of Freeze-Dried Cell Walls Revealed Structural Changes in Cell Walls after Heat Activation

Another technique, X-ray diffraction analysis was applied to cell wall preparations to evaluate changes of the cell wall after heat treatment. Cell wall preparations of dormant or activated spores were prepared by breaking ascospores with glass beads (see Supplementary Figure S1). Spectra were recorded to observe possible changes in the structure of the outer cell walls, part of the samples were treated with SDS after they had been washed with ultra-pure water and some of the SDS-treated samples were additionally treated with 2 N KOH. All the cell walls were freeze dried and the hereby obtained powder was used to record X-ray spectra (Figure 1f–h). Cell wall preparations of non-heat-activated ascospores show a similar spectrum, either after SDS-treatment and subsequent KOH extraction. These spectra only have a diffuse dark band near the center of the photographic film, indicating the presence of (crystalline) chitin. The spectra of the cell walls from activated ascospores (indicated by arrows) show more defined line patterns in all cases. This indicates that the material has a more crystalline structure ranging from numerous lines in washed cell walls (possibly small proteins) that disappear upon extraction with SDS and KOH. A possible α1–3 glucan spectrum, visible as two inner lines in Figure 1g is present in in SDS washed cell walls, that disappear after a subsequent KOH treatment. The chitin spectrum is weak, indicating that after heat activation this compound can be extracted more easily after KOH treatment. Probably, chitin is prevented from crystallisation which can make it more vulnerable for enzymatic attack necessary for growth. These observations suggest that ascospore cell walls have changed markedly upon heat treatment.

### 3.4. Protein Release during Heat Activation

**Figure 2:**
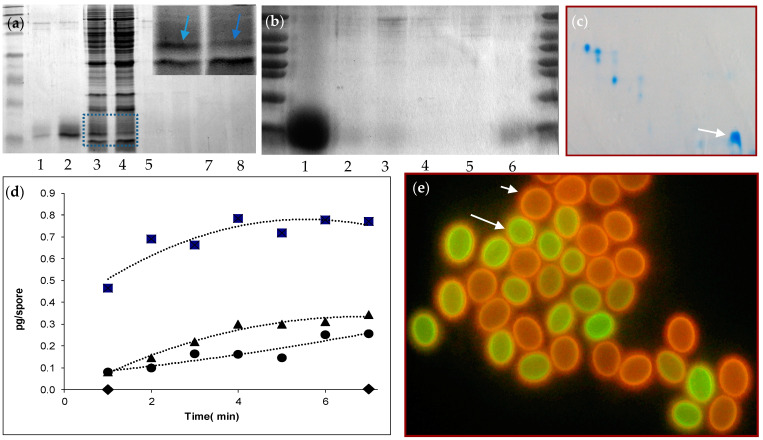
During heat activation a small protein is released from the cell wall, which becomes permeable after heat activation. (**a**,**b**) SDS-PAGE gels of supernatants and cell free extracts of dormant and activated ascospores. The two lower marker bands are 6.5 and 16.5 kD, respectively. (**a**) (1) Supernatant from dormant spores and (2) heat-activated spores. (3) Cell free extract of broken, dormant spores and (4) heat-activated spores. The box (inset) indicates a decrease of a protein (designated as blue arrows) corresponding to the one released after heat activation. (5) Supernatants of heated (7 min at 85 °C) cell wall preparations of dormant spores and (6) activated ascospores. (7) Cell wall preparations after treatment in 2% SDS of dormant or activated (8) spores. (**b**) (1) Direct gel-electrophoresis of dormant spores boiled in sample buffer. (2) Supernatant of activated spores boiled in sample buffer (SB). (3) As lane (2), but with slightly older spores (45 days instead of 40). (4,5) show the same as (2,3) but with cell walls of activated spores. (6) shows supernatant of dormant ascospores. (**c**) 2 D-gel electrophoresis illustrates the abundance of the small protein within the context of all cell proteins in a cell free extract originating from broken ascospores (see white arrow). (**d**) Measurement of released protein of ascospore suspension after heat treatment. The different lines indicate the amount of protein release as calculated for a single spore (pg/spore) after a 1 to 7 min activation at 22 °C (◆), 65 °C (●), 75 °C (▲) and 85 °C (■). (**e**) Ascospores of *T. macrosporus* exhibit a red autofluorescence during fluorescence microscopy (short arrow). After a short, 1 min heat-activation, part of the spores shows a green staining of carboxyfluorescein inside the cell wall (long arrow).

The changes in the cell wall structure could be accompanied by the release of components, including proteins, during a heat treatment. Release of protein was assessed by heating the ascospores to 85 °C for 7 min and evaluating proteins on SDS-PAGE gels (Figure 2a–c). Alternatively, ascospores were heated in sample buffer. A very broad and dominant band between the 6.5 and 16.5 kD marker was observed. In other experiments, different representations of bands in this region were observed. One of the most prominent forms was a single band with a bit of a smear around it (Figure 2a(2),b(1)). In one case more than one band was observed (Supplementary Figure S2). Some protein was already present in the supernatant before activation (Figure 2a(1),b(6)). Cell free extracts of broken dormant or heat-activated ascospores with the supernatant removed after heat treatment before breaking of the cells, show a decrease of the density of one protein band in the low mol. weight area of the gel that corresponds to the low weight band (box and inset, Figure 2a(3,4)). If dormant and activated ascospores were washed thoroughly and subsequently broken, the proportion of this protein is not visible on gel (Figure 2a(5,6)) as was after SDS treatment of the cell wall preparations (Figure 2a(7,8)). If cell walls of dormant ascospores were broken and boiled in sample buffer and applied to gel, some protein was visible (Figure 2b(2,3)), which was not visible when heat activation was applied to the ascospores (Figure 2b(4,5)). 

To quantify protein release, a BCA protein assay was conducted (Figure 2d). The ascospores were kept at different temperatures in buffer for 1–7 min and samples were taken for protein release at regular time intervals. Afterwards supernatant was obtained by spinning the spores down shortly and protein was measured. At increasing temperatures, larger amounts of protein are released. At room temperature virtually no protein was released. At 65 °C and 75 °C averages of 0.3 and 0.4 pg/spore were released within 7 min. At 85 °C an increase in the amount of released protein was observed, with an average of 0.8 pg/spore. It was also observed that the bulk of the protein in this case was released within the first two minutes of activation.

### 3.5. Staining of Ascospores with Carboxyfluorescein

The changes observed in the ascospore cell wall after a heat treatment may result in an altered permeability to water or other molecules. We used fluorescein as an indicator of cell wall permeability. Ascospores of *T. macrosporus* exhibit red autofluorescence of the outer layer of the cell wall (Figure 2e). Dormant spores incubated in 3.25 mM fluorescein for 15 min (30 °C, 160 rpm) did not show green staining (short arrow). However, after heat activation in the presence of fluorescein, a bright green fluorescent staining inside the cell wall, at the inner side of the autofluorescence, was clearly visible (Figure 2e, long arrow). In another experiment, ascospores were heated for 0, 10, 20, 40 and 80 s. Corresponding numbers of fluorescein-stained cell walls were 2%, 5%, 4%, 8.5%, 55%. This indicates that changes in permeability occur in the cell wall appear after 40 s.

Heat activation of ascospores is followed by a sudden shedding of the outer cell wall after 3.5–4.5 h. This process is described earlier [16,17] and depicted in Supplementary Figure S3. After this shedding process, the green fluorescent staining was accumulating in the buffer around the spores, slowly increasing the background fluorescence. This indicates that dye was not taken up into the cytoplasm, but permeability of the cell wall was the sole cause of staining.

Two batches of ascospores were heat treated (150 s at 85 °C) in the presence of dye and gently inoculated on a thin agar layer on an objective slide. Directly, fluorescence microscopy was done and the fluorescence inside the cell wall assessed via Adobe Photoshop by measuring the maximal pixel intensity (as expressed in arbitrary units) along a line fragment. Then, the slides and ascospores were kept in a moist environment for 6 or 7 h respectively and studied again with light microscopy. The ascospores that had exhibited shedding of the outer cell wall showed highly significantly increased green staining of the cell wall immediately after heat activation. In one experiment with 27 non-shedded and 25 shedded cells, the mean staining intensities were 22.4 and 30.2, respectively (*p* < 0.001 according to T-tests assuming equal variances). In a replicate experiment with 28 non-shedded and 18 shedded cells, the mean staining intensities were 23 and 33.9 (*p* < 0.001). This suggest that permeability of the outer cell is related to activation and germination of the spores.

### 3.6. Analysis of the ICARUS Gene

Since our results pointed to a single protein as being the major component of the cell wall released upon heating, we turned our attention to identifying this protein. In order to obtain the full sequence of the protein, which we designated as ICARUS*, it was N-terminally sequenced using the Edman degradation method. Fractions containing the proteins were separated by means of PAGE. When blotting occurred at 100 V for 1 h no protein could be detected. It was assumed that the protein migrated too fast through the membrane. Reduction to 60 V for 4.5 min resulted on retention of the protein on the PVDF membrane. The lowest of the four bands was cut out and sent for N-terminal sequence. The first ten amino acids were determined resulting in the sequence QPTLTDDADY. Based on this sequence, degenerate primers were designed for a RT-PCR on total RNA isolated from *T. macrosporus* cultures. A DNA fragment putatively encoding ICARUS was obtained, cloned and sequenced.

**Figure 3:**
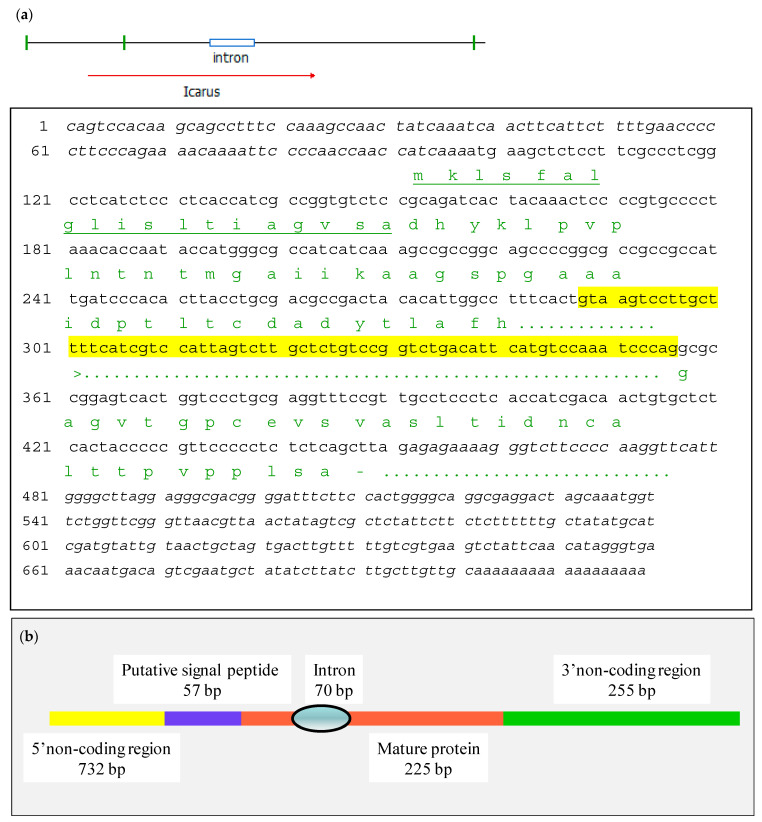
Detailed description of the ICARUS gene (**a**). Sequence of the ICARUS coding region beginning at the transcription starting point. Protein sequence is shown below the exonic regions, with the putative signal peptide underlined. The only intron is highlighted in yellow. The amino acids are shown in green. (**b**). Summary of the ICARUS transcript structure.

The sequence revealed a stretch of 404 bp including the degenerate primer and down to the polyA tail. The open reading frame in continuation of the degenerate primer encoded 45 aa residues (see for the structure of the gene Figure 3). In order to determine the amino terminal part of the protein, 5′Race PCR was done using a reverse primer based on the obtained cDNA sequence. After PCR, a fragment was isolated of 627 bp which was cloned and sequenced (Supplementary Figure S4a). The fragment contains a methionine codon in frame with t the determined amino terminus and added 49 aa residues upstream of it. Analysis by SignalP-5 (CBS, central for biological sequence analysis) revealed the presence of an expected signal peptide for secretion of 19 aa residues bringing the size of the mature protein back to 75 aa residues with an estimated molecular mass of 7526 Da. The cDNA contained a 5′UTR of 97 bp and a 3′UTR of 250 bp (see Figure 3). To assess the presence of introns a similar PCR was done on chromosomal DNA from *T. macrosporus* using primers plcfw1 and playracer. The amplified sequence showed the presence of one intron in the coding sequence of Icarus (Supplementary Figure S4b and Figure 3). A summarizing scheme of the gene structure is shown in Figure 3b showing one intron and a putative signaling sequence. The precise base to base structure of the gene is shown in Figure 3a.

### 3.7. Expression of the ICARUS Gene

**Figure 4:**
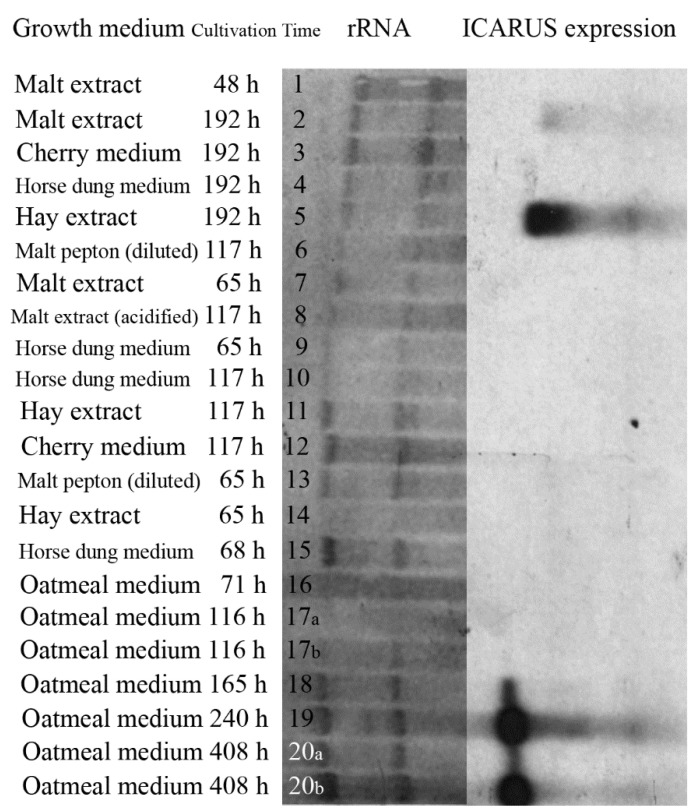
Expression of the ICARUS Gene. *T. macrosporus* has been cultivated on a large numbers of different growth media. The left part of the blot indicates the different amounts of ribosomal RNA present; the right part indicates the ICARUS positive expression signals that are observed after growth on the media designated with the labels 2, 5, 18, 19, 20a and 20b. These are all samples of cultures that show ascomata (fruit body) formation.

If ICARUS expression is related to the formation of ascomata and ascospores, this would provide evidence for its role in ascospore development and dormancy. *T. macrosporus* was cultivated on different growth media some of them conducive for ascomata production. RNA was isolated and used on a Northern blot analysis. The earlier obtained cDNA fragment was used as probe to study gene expression. Notably, the specific mRNA of the ICARUS gene was identified in cultures grown on oatmeal agar after one week of growth when ascomata are formed and increasing at a later time point when more fruiting bodies and ascospores were formed (Figure 4). In addition, mRNA was very strongly present in cultivated fungus on hay extract medium that very strongly provokes ascoma formation, while less expression on malt extract medium was observed, where less ascomata were observed. All other growth conditions (see Supplementary Table S2) that exhibited only formation of hyphae, aerial hyphae and conidiophores did not show hybridization. For example, horse dung medium or cherry medium did not support sexual fruit body formation and did not result in notable expression even when rRNA levels indicated higher RNA concentration. Further, no expression was visible when cultures were incubated for 117 h or lower in all cases. Taken together, ICARUS expression could be linked to the formation of mature ascomata and henceforth ascospores.

### 3.8. Obtention of an ICARUS Deletion Mutant

If ICARUS is an important protein functioning in the constitutive dormancy of ascospores, deletion of the gene would lead to ascospores that have exhibit differences in ascospore activation. A deletion construct was as a tool to generate a functional inactivation of the ICARUS gene by homologous recombination. This imposed the need of extra sequences flanking the region to be deleted. In order to obtain a larger fragment of the region around the ICARUS locus, the cDNA was used as a probe in a Southern Blot experiment (Supplementary Figure S4c). This experiment showed that only one copy of the gene was present in the genome and revealed BglII as a suitable restriction enzyme to attempt to amplify the fragments flanking the coding region. For this task, genomic DNA was cut with BglII and ligated following the splinkerette’s protocol. PCR primers were used, which annealed at the outer regions of the cDNA fragment and would be elongated outwardly. Only for the PCR using a reverse primer on the 5′UTR, a 900 bp DNA fragment was obtained, cloned and sequenced. It contained a short stretch of the upstream ICARUS cDNA, which confirm the specificity of the fragment. This resulted in the obtention of 870 bp of the upstream region of the gene. Since no downstream flank to the cDNA could be isolated, part of the coding region was used as an “homologous flank”. In this way the deletion would consist in eliminating the TATA box of the promoter down to the region encoding the amino terminus of the protein, including the signal peptide.

Deletion construct pIcardel (Supplementary Figure S5) was transformed to protoplasts of *T. macrosporus* and hygromycin selection was applied. Plenty of transformants were isolated. Screening at the protein level was considered, but this approach involved the production of mature ascospores, their harvesting, activation and subsequent analysis of supernatants on SDS-PAGE. It was then decided to screen for the deletion by means of Northern blot analysis using as probe a fragment of the region to be deleted. Two transformants were found to lack the band corresponding to the ICARUSs transcript, which were then dubbed 47 C and X and were analyzed further.

### 3.9. Characterization of the ICARUS Deletion Mutants

**Figure 5:**
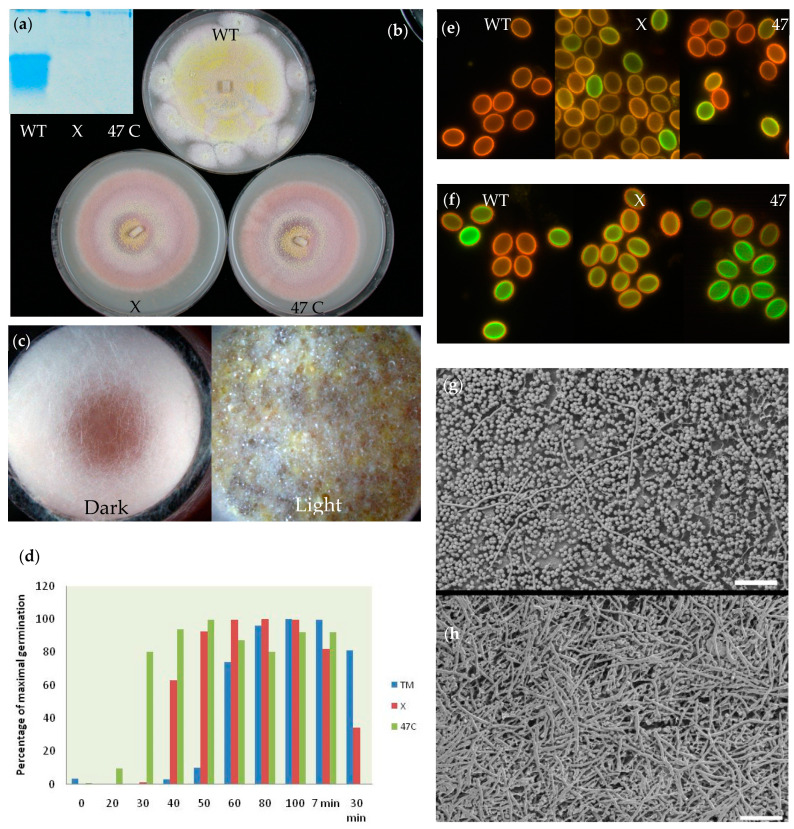
Properties of ICARUS mutants (**a**–**h**) (**a**) No protein was observed in the supernatant of mutant ascospores after heat activation. (**b**) Agar surfaces show different morphology of colonies of the wildtype in comparison with the mutant strains after 8 days of growth. Mutant colonies show later formation of ascomata (visible as yellow dots) and exhibit a red color due to a pigment which is also released into the agar. (**c**) Growth of mutant 47 C on oatmeal medium in the dark does not show the presence of ascomata after 8 days of incubation. Microtiter plates were wrapped in tin foil and kept in a closed box. (**d**) Colony count on plates that are inoculated with 400 ascospores of wild type and mutant strains that are heat treated for very short time periods (20–100 s) at 85° C. Note that survival of the mutant ascospores is less after 30 min of heat treatment. TM = *Talaromyces macrosporus* wild type strain; X and 47 C are the names of the mutant strains (**e**) Autofluorescence (red) and carboxy fluorescein staining (green) of the cell wall of ascospores without heat treatment. Mutant strains show spontaneous green staining of part of the spores, indicating a more permeable cell wall. (**f**) Staining of ascospore after a 1 min heat treatment at 85 °C, mutant ascospores all show a ring of staining, while part of the wild type ascospores do not exhibit green staining. (**g**) Numerous ascospores of the wildtype after 1 min of heat treatment and 19 h of incubation on malt extract medium at 25 °C. (**h**) Mutant strain 47 C show abundant formation of hyphae at the same time indicating that much more ascospores have germinated. Bar in (**g**,**h**) is 50 µm.

Ascospores of both mutant strains did not show any presence of a low-weight protein in supernatant of ascospores after heat activation (Figure 5a). The colony appearance of both mutants was markedly different from the wildtype. Both mutant strains showed delayed ascomata formation and the release of a red pigment in oatmeal agar (Figure 5b). Remarkably, Figure 5c shows that strain 47C was not able to form ascomata in the dark on oatmeal medium after 8 days of incubation, while it does in the light. The wildtype strain was able to form these structures in both light and dark, albeit with more red pigment and lower densities in the latter condition. The morphology of the ascospores of wildtype and the mutants was not different as judged by scanning electron microscopy (Supplementary Figure S6) and also at high magnification using cryoplaning, at first sight, no differences in the structure of the cell wall were observed (Supplementary Figure S7). Colony formation after a 1 min treatment of ascospore suspension that was subsequently spread out on agar surfaces resulted in a much higher number of germinated mutant cells and strain 47 C exhibited germination of the majority of the cells in 30 s time (Figure 5d). Surprisingly, ascospores of both mutant strains showed much less germination after a heat treatment of 30 min indicating that the heat resistance of ascospores might be affected by the absence of the ICARUS protein. Ascospores of the mutants stained with carboxy-fluorescein, showed already staining inside the cell wall of some spores even without heat treatment (Figure 5e). After a 1 min heat treatment at 85 °C, part of the ascospores of the wild type showed staining. Both mutants showed staining in all cells (Figure 5f). Cryo-scanning electron microscopy showed extensive hyphae formation from mutant strain 47 C ascospores after following a 1 min heat treatment and 19 h of cultivation on agar. The wildtype showed in the majority of the cases, non-germinated ascospores and only sporadic hyphae (Figure 5g,h). All these observations suggest that the small ICARUS protein has a function in ascospore dormancy and hints on a further functioning during ascomata formation.

## 4. Discussion

Dormant fungal cells exhibit a lowered metabolism, accumulate protective compounds and delineate their protoplasts with a thick outer cell wall. Two types of dormancy are mentioned in literature including endogenous (or also called constitutive) dormancy [9,30] or exogenous (or environmental) dormancy.

According to [9] and later discussed by [30,31], dormancy is: “Any rest period or reversible interruption of the phenotypic development of the organism.” Constitutive or endogenous dormancy is: “A condition wherein development is delayed due to an innate property of the dormant stage such as a barrier to the penetration of nutrients, a metabolic block, or the production of a self-inhibitor”. Exogenous dormancy is: “A condition wherein development is delayed because of unfavorable chemical or physical conditions of the environment”.

Ascospores of *T. macrosporus* do only germinate in very low, if any, numbers when present in or on a rich nutrient liquid or agar medium [16] and therefore can be defined as constitutively dormant spores. It would be of interest to evaluate in detail, if these spores do not germinate at all in these media and that sparsely present conidia or hyphal cells do account for the low colony counts observed. Further, these cells are triggered into massive synchronized germination after being provoked by an extreme trigger, which, presumably, takes the barrier for germination away. The nature of the cause of delayed germination is unknown. The data in this study provide evidence that the thick, multi-layered (see also [20]) cell wall of *T. macrosporus* ascospores changes after a heat treatment including: (i.) a change in its structure as shown with EPR and X-ray diffraction; (ii.) a release of an abundant protein into the supernatant after heat activation, which is proportional to the extent of heat activation; (iii.) a change in the permeability of the cell wall as judged by fluorescence studies in which staining of the interior of the cell wall correlated with individual germination of individual ascospores.

**Figure 6:**
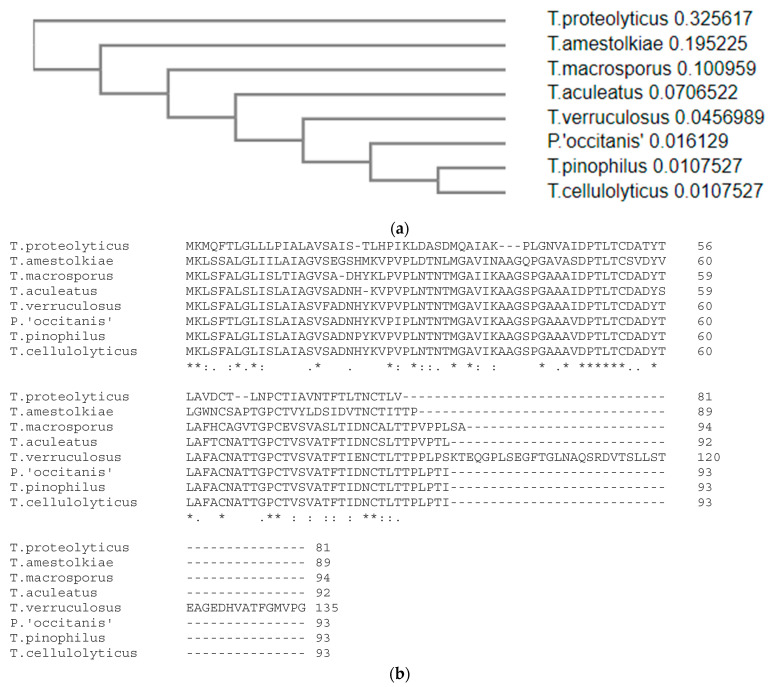
Putative ICARUS sequences in other *Talaromyces* species (**a**). Blast search of different genomes. (**b**) A similarity tree based on these sequences.

The gene encoding the protein, dubbed ICARUS, was studied in detail and was expressed under growth conditions that showed intense ascomata (fruit body) formation and therefore ascospore formation. It is a small 7–14 kD protein that is not comparable to anything we know (this work was initiated long ago and has now been approached as a “cold case”). At the time of isolation of ICARUS, no homologies could be found in the DNA or protein databases. Recently, new blast jobs were attempted and this action rendered 7 orthologues in 6 other members of the genus *Talaromyces* and in a *Penicillium* species (Figure 6). The predicate “hypothetical protein” given to these 7 proteins stressed the importance of publishing all data accumulated on ICARUS. Especially when attending at its properties and its role in the interesting process it is involved in. 

Mutant strains show a delayed ascomata formation, release of pigments into the agar medium or liquid medium, changes in the formation of fruiting bodies in the dark and a decreased dormancy of the spores, which need a shorter heat treatment for activation. All these observations suggest that the protein plays a role in dormancy and permeability of the ascospore cell wall. It is not yet clear if ICARUS is an integral part of the entire cell wall or present as an outer layer, deposited on the ascospores during maturation within the ascus mother cell. The changes in structure and permeability of the cell wall after release of the protein suggest that the protein might be related to the integral structure of the ascospore cell. Electron microscopical techniques applied at high magnification, however, do not show marked differences between the wild type and mutant cell wall structure or the nature of the ornamentation, indicating that the change is one on a very small scale (Supplementary Figures S6 and S7). A high temperature is needed to provoke the release of the protein, with ascospores that are repetitively washed in buffer, indicating that the protein is not very loosely correlated to the ascospore.

The ICARUS mutant strains release red pigment in the growth medium. The orange/red color of ascospores is a hallmark of *T. macrosporus* and the absence of the protein may affect pigment localization in the cell wall during early ascoma formation, which is accompanied with loss of pigment in the medium as if it could be less effectively incorporated or retained in ascomata and ascospores. This is possibly the red pigment mitorubrin, but this fungus also forms and abundant secondary metabolite named duclauxin [32]. The monomer of duclauxin has some resemblance to the melanin monomers and could, based on its structure, very well fluoresce redly and may have a function in cell wall structure. However, there are quite some species that show constitutive dormancy including unpigmented species as *Aspergillus fischeri* (with a neosartorya morph) and *Paecilomyces niveus* (with a byssochlamys morph). If constitutive dormancy is conveyed by abundant small proteins as ICARUS, pigment binding must be different. These are aspects that needs to be studied further. These unpigmented species also show extensive heat resistance, equal to *T. macrosporus* [3]. A tantalizing observation even suggests that the function of the protein expands to signaling during fruit body formation as ascomata are not formed in mutant strains in the dark. For instance, in playing a role in light sensing via the pigment that is signaled downstream to evoke the onset of sexual spore formation.

There is clearly more research needed on this including the following research questions: (i.) Is a similar mechanism observed with other constitutively dormant ascospores [2,3,4,6,15,20]? These include the unpigmented species as stated above. (ii.) Are there other factors involved as there is still some heat activation needed? Analysis of cell wall preparations by means of Fourier Transformed Infrared Spectroscopy (FTIR) show that the CH asymmetric stretching vibrations, that originate from CH_2_ and CH_3_ of hydrocarbons, show a marked change between 60 and 80 °C, which are temperatures near to those that activate the ascospores. This could mean that temperature-related changes occur in the carbohydrate backbone of cell walls (Supplementary Figure S8 and Table S3, Appendix A). (iii.) Is impermeability of the cell wall the main mechanism in constitutive dormancy? Sussman and coworkers suggest [9] that impermeability is not leading in dormancy of *Neurospora* ascospores. Indeed, the cytoplasm of ascospores of *Talaromyces* and *Aspergillus* species with a neosartorya morph is a fluid, indicating that water is present in the cell [4,17]. This might point towards a role of ICARUS that is more complex as solely a barrier function, but this aspect surely needs more detailed research.

However, upon heat activation the thick outer cell wall must be shed [17,20] (as illustrated by Supplementary Figure S3 in this manuscript) followed by a strong increase in respiration, which was not measurable in a dense suspension of ascospores before this shedding stage [16]. The inner cell exhibits swelling and subsequent germ tube formation leading to the formation of a mycelium. There, the transformation of a highly stress-resistant dormant spore towards a growing vegetative cell is completed.

## Supplementary Materials

The following are available online at https://www.mdpi.com/2309-608X/7/3/216/s1.

## Appendix A

Supplementary Material and Methods FTIR (Fourier Transformed Infrared Spectroscopy)

Small amount of dry cell wall (CW) material (dried 4 d, 3%RH) was placed between two circular CaF2 windows (2 × 13 mm) without rubber ring and mounted into a temperature-controlled cell. Infrared spectra of dry cell wall preparations were obtained with a Perkin-Elmer series1725 FTIR spectrometer equipped with an external beam facility to which a Perkin-Elmer IR-microscope was attached. The microscope was equipped with a narrow band mercury-cadmium-telluride liquid nitrogen-cooled IR-detector. The samples between two CaF2 windows were tightly mounted into a temperature-controlled bras cell that was cooled by liquid nitrogen. The temperature of the cell was regulated by a computer-controlled device that activated a liquid nitrogen pump in conjunction with a power supply for heating the cell. The temperature of the sample was recorded separately using two PT-100 elements that were located very close to the sample windows. The optical bench was purged with dry CO2-free air. Spectra were recorded starting with the lowest temperature with a scanning rate of 1.5 °C/min. The acquisition parameters were: 4 cm^−1^ resolution, 32 co-added interferograms, 3500–1000 cm^−1^ wavenumber range. Spectral analysis and display were carried out using the Infrared Data Manager Analytical software, version 3.5 (Perkin–Elmer). The temperature-induced changes in dry were monitored by observing the position of the bands Amid I (around 1662 cm^−1^), CH asymmetric stretch (around 2915 cm^−1^) and O-H stretching vibrations (around 3339 cm^−1^).

Amid I is mainly associated with the C=O. stretching vibration (70–85%) and is directly related to the protein backbone conformation. The CH asymmetric stretching vibrations originate from CH2 and CH3 of hydrocarbons. OH stretching vibrations in a cell wall relates to bonded and non-bonded –O–H groups.

## References

[1] Dijksterhuis J., Dijksterhuis J., Samson R.A. (2007). Heat-resistant ascospores. Food Mycology: A Multifaceted Approach to Fungi and Food.

[2] Dijksterhuis J. (2019). Fungal spores: Highly variable and stress-resistant vehicles for distribution and spoilage. Food Microbiol..

[3] Wyatt T.T., Van Leeuwen M.R., Golovina E.A., Hoekstra F.A., Kuenstner E.J., Palumbo E.A., Snyder N.L., Visagie C., Verkennis A., Hallsworth J.E. (2015). Functionality and prevalence of trehalose-based oligosaccharides as novel compatible solutes in ascospores of *Neosartorya fischeri* (*Aspergillus fischeri*) and other fungi. Environ. Microbiol..

[4] Wyatt T.T., Golovina E.A., Van Leeuwen M.R., Hallsworth J.E., Wösten H.A.B., Dijksterhuis J. (2015). A decrease in bulk water and mannitol and accumulation of trehalose and trehalose-based oligosaccharides define a two-stage maturation process towards extreme stress resistance in ascospores of *Neosartorya fischeri* (*Aspergillus fischeri*). Environ. Microbiol..

[5] Houbraken J., Varga J., Rico-Munoz E., Johnson S., Samson R.A. (2008). Sexual reproduction as the cause of heat resistance in the food spoilage fungus *Byssochlamys spectabilis* (Anamorph *Paecilomyces variotii*). Appl. Environ. Microbiol..

[6] O’Gorman C., Fuller H.T., Dyer P.S. (2009). Discovery of a sexual cycle in the opportunistic fungal pathogen Aspergillus fumigatus. Nature.

[7] Hayer K., Stratford M., Archer D.B. (2013). Structural features of sugars that trigger or support conidial germination in the filamentous fungus *Aspergillus niger*. Appl. Environ. Microbiol..

[8] Hayer K., Stratford M., Archer D.B. (2014). Germination of *Aspergillus niger* conidia is triggered by nitrogen compounds related to L-amino acids. Appl. Environ. Microbiol..

[9] Sussman A.S., Halvorson H.O. (1966). Spores, Their Dormancy and Germination.

[10] Beuchat L.R. (1986). Extraordinary heat resistance of *Talaromyces flavus* and *Neosartorya fischeri* ascospores in fruit products. J. Food Sci..

[11] Reyns K.M., Veraverbeke E.A., Michiels C.W. (2003). Activation and inactivation of *Talaromyces macrosporus* ascospores by high hydrostatic pressure. J. Food Prot..

[12] Dijksterhuis J., Teunissen P.G. (2004). Dormant ascospores of *Talaromyces macrosporus* are activated to germinate after treatment with ultra-high pressure. J. Appl. Microbiol..

[13] Tournas V. (1994). Heat-resistant fungi of importance to the food and beverage industry. Crit. Rev. Microbiol..

[14] Dos Santos J.P.L., Samapundo S., Biyikli A., Van Impe J., Akkermans S., Höfte M., Nji Abatih E., Sant’Ana A.S., Devlieghere F. (2018). Occurrence, distribution and contamination levels of heat-resistant moulds throughout the processing of pasteurized high-acid fruit products. Int. J. Food Microbiol..

[15] Berni E., Tranquillini R., Scaramuzza N., Brutti A., Bernini V. (2017). *Aspergilli* with *Neosartorya* type ascospores: Heat resistance and effect of sugar concentration on growth and spoilage incidence in berry products. Int. J. Food Microbiol..

[16] Dijksterhuis J., Van Driel K.G., Sanders M.G., Molenaar D., Houbraken J.A., Samson R.A., Kets E.P. (2002). Trehalose degradation and glucose efflux precede cell ejection during germination of heat-resistant ascospores of *Talaromyces macrosporus*. Arch. Microbiol..

[17] Dijksterhuis J., Nijsse J., Hoekstra F.A., Golovina E.A. (2007). High viscosity and anisotropy characterize the cytoplasm of fungal dormant stress-resistant spores. Eukaryotic Cell.

[18] Kikoku Y. (2006). Heat activation characteristics of *Talaromyces* ascospores. J. Food Sci..

[19] Kikoku Y., Tagashira N., Gabriel A.A., Nakano H. (2009). Heat activation of *Neosartorya* and *Talaromyces* ascospores and enhancement by organic acids. Biocontrol Sci..

[20] Wyatt T.T., Wösten H.A.B., Dijksterhuis J. (2013). Fungal spores for dispersion in space and time. Adv. Appl. Microbiol..

[21] Novodvorska M., Stratford M., Blythe M., Wilson M.J., Beniston R.G., Archer D.B. (2016). Metabolic activity in dormant conidia of *Aspergillus niger* and developmental changes during conidial outgrowth. Fungal Genet. Biol..

[22] Dijksterhuis J., Samson R.A., Hocking A.D., Pitt J.I., Samson R.A., Thrane U. (2006). Activation of ascospores by novel food preservation techniques. Advances in Food Mycology.

[23] Frisvad J.C., Filtenborg O., Samson R.A., Stolk A.C. (1990). Chemotaxonomy of the genus *Talaromyces*. Antonie van Leeuwenhoek.

[24] Sietsma J.H., Wessels J.G.H. (1977). Chemical analysis of the hyphal wall of *Schizophyllum commune*. Biochim. Biophys. Acta.

[25] O’Farrell P.H. (1975). High resolution two dimensional electrophoresis of proteins. J. Biol. Chem..

[26] Schuren F.H.J., Harmsen M.C., Wessels J.G.H. (1993). A homologous gene-reporter system for the basidiomycete *Schizophyllum commune* based on internally deleted homologous genes. Mol. Gen. Genet..

[27] Devon R.S., Porteous D.J., Brookes A.J. (1995). Splinkerettes—Improved vectorettes for greater efficiency in PCR walking. Nucleic Acids Res..

[28] Murray F.R., Llewellyn D.J., Peacock W.J., Dennis E.S. (1997). Isolation of the glucose oxidase gene from *Talaromyces flavus* and characterisation of its role in the biocontrol of *Verticillium dahlia*. Curr. Genet..

[29] Crous P.W., Verkleij G.J.M., Groenewald J.Z., Houbraken J. (2015). Fungal Biodiversity.

[30] Cochrane V.W. (1974). Dormancy in spores of fungi. Trans. Am. Microsc. Soc..

[31] Feofilova E.P., Ivashechkin A.A., Alekhin A.I., Sergeeva Y.A. (2012). Fungal Spores: Dormancy, Germination, Chemical Composition and Role in Biotechnology (Review). Appl. Biochem. Microbiol..

[32] Yilmaz N., Visagie C.M., Houbraken J., Frisvad J.C., Samson R.A. (2014). Polyphasic taxonomy of the genus *Talaromyces*. Stud. Mycol..

