# Supplementary material for: Abundant Small Protein ICARUS Inside the Cell Wall of Stress-Resistant Ascospores of Talaromyces macrosporus Suggests a Novel Mechanism of Constitutive Dormancy
Source: J Fungi (Basel). 2021 Mar 17;7(3):216. doi: 10.3390/jof7030216 (PMC8002430; doi:10.3390/jof7030216)
Supplement: Supplementary file 1 [file jof-07-00216-s001.pdf]

Supplementary Materials: The following are available online at www.mdpi.com/xxx/s1,

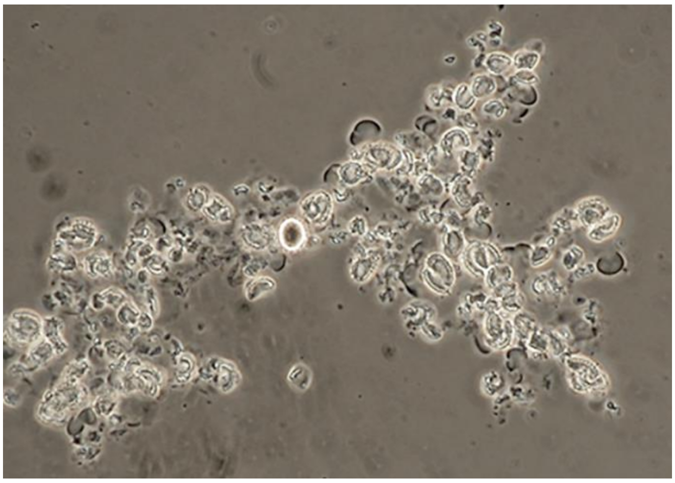

Supplementary Figure S1. Broken ascospores after vortexing with glass beads

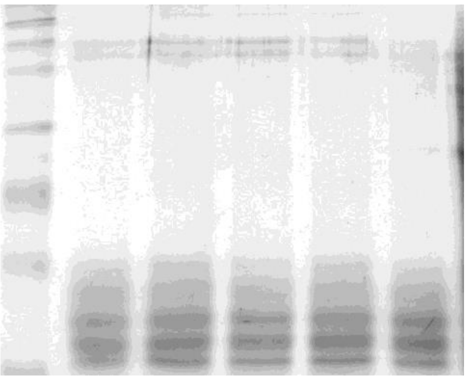

Supplementary Figure S2. Gel with protein in released supernatant showing several bands of small protein.

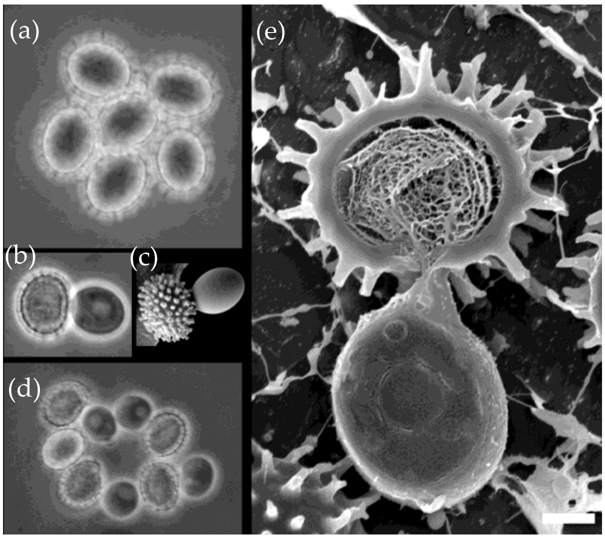

(c)

Supplementary Figure S3. Activated ascospores shed their outer cell walls a-c) Dormant ascospores exhibit a dense appearance with a high refractive index as observed with phase contrast microscopy (a). After heat activation, the inner cell jumps out of the outer cells wall (b, see [16], the emptied outer cell wall is visible and its thickness clearly visible. (c) cryoSEM of shedded outer cell wall and inner cell. d) Four ascospores that have shedded and one dormant spore in between. e) Cryoplaning, a SEM technique that involves cutting of frozen cells, shows the emptied outer cell wall and the inner spore, containing organelles, outside it (see also [17]). Bar =1 μm.

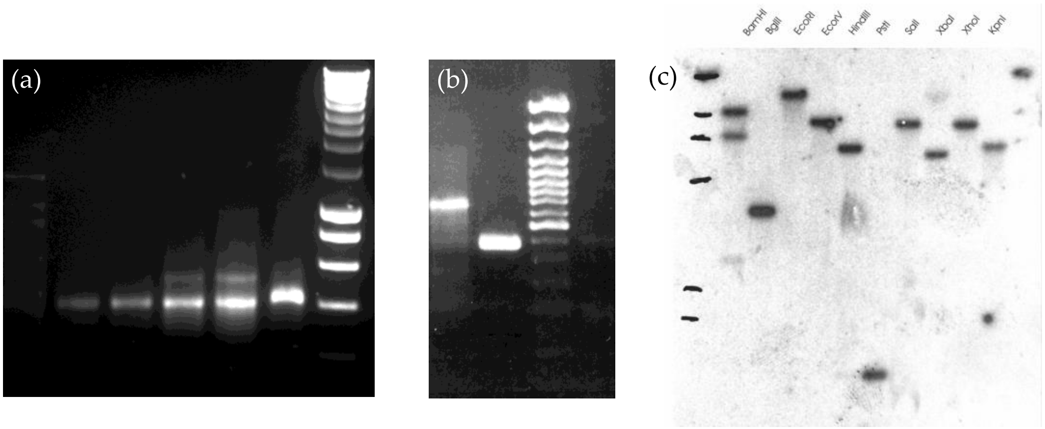

Supplementary Figure S4. (a). PCR of ICARUS fragment using different mixtures of compounds (b) Fragment of ICARUS originating from chromosomal DNA containing the intron (line 1) and originating from cDNA originating of the mRNA pool (line 2). (c) Southern Blot indicating hybridization to one fragment containing the entire ICARUS gene by several restriction enzymes indicating that only one copy of the gene is available in the genome.

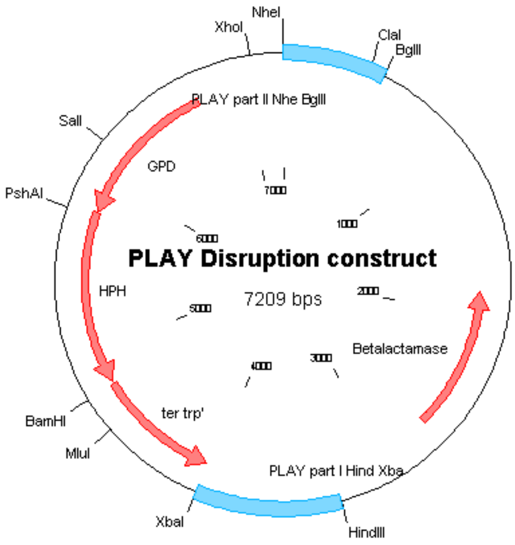

Supplementary Figure S5. Construct used for the preparation of a functional deletion mutant.

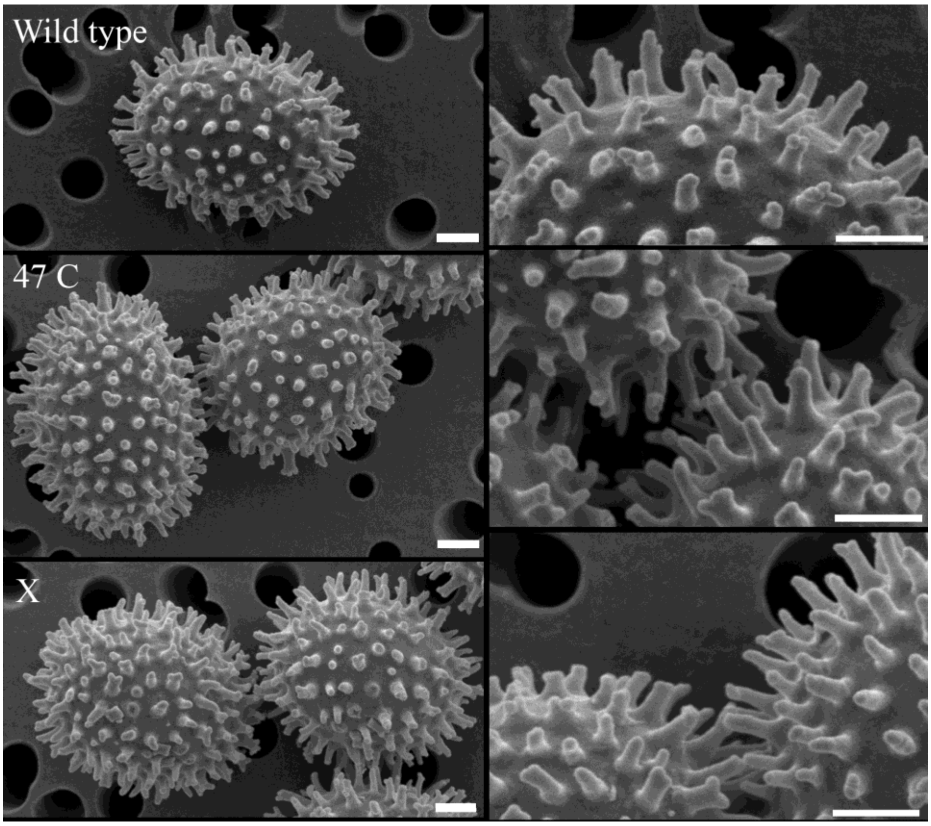

Supplementary Figure S6. Scanning electron microscopy on air dried ascospores of the wildtype strain and the two mutant strains. No differences in ornamentation are visible. Bars= 1μm.

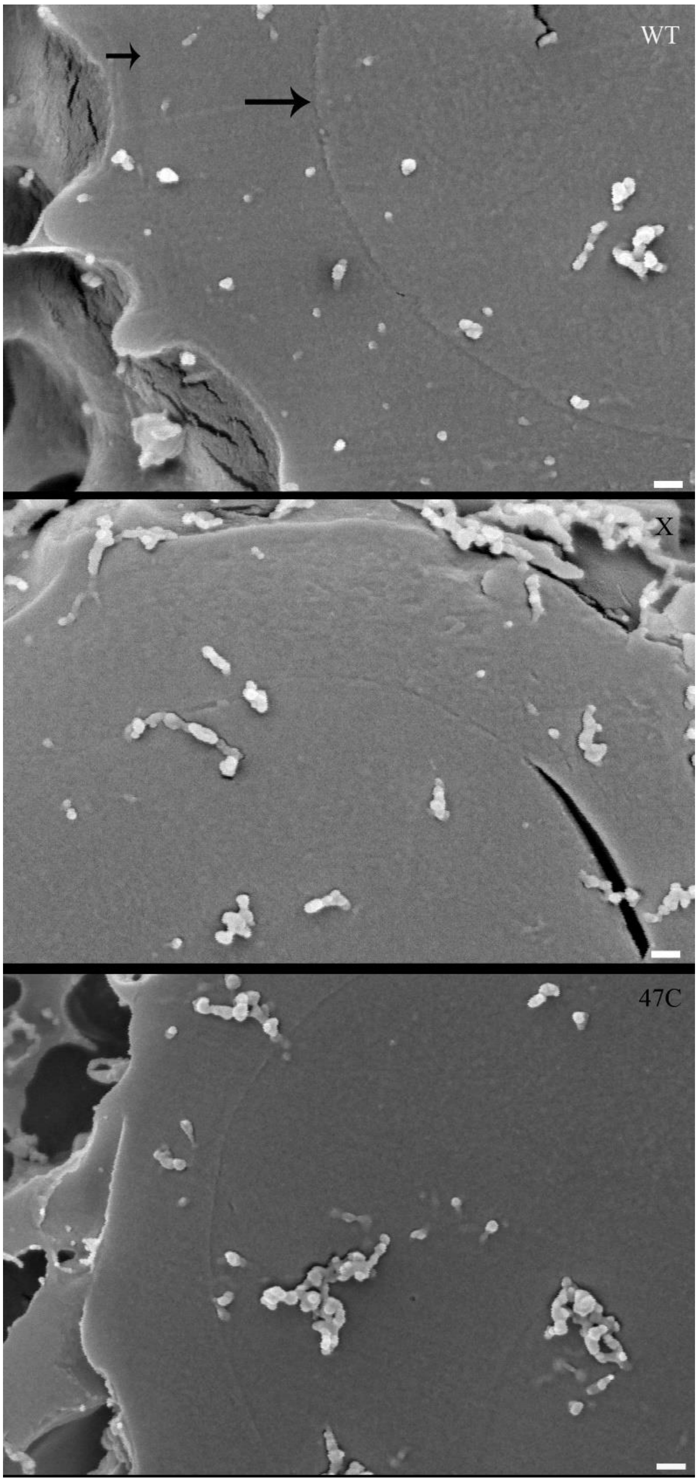

Supplementary Figure S7. Cryoplaning of ascospores at high magnification shows the dimension of a very thick cell wall that encompasses the cell interior (large arrow). In addition, an extra wall layer could be discerned next to the ornamentation (small arrow). No clear differences were visible between the WT and mutant cell walls. Bars= 100 nm.

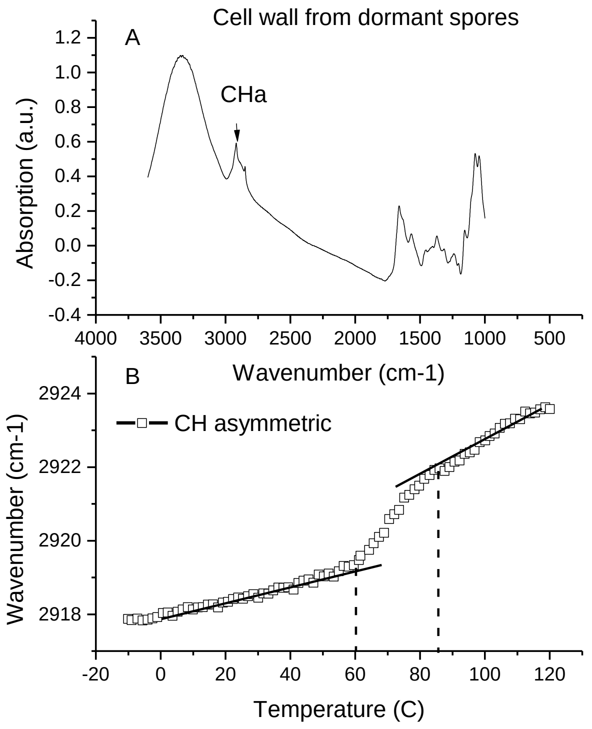

Supplementary Figure S8. A – FTIR spectrum from dried cell wall preparations from dormant spores (CHa - CH2 and CH3 asymmetric stretching vibrations band); The CH asymmetric stretching vibrations originate from CH2 and CH3 of hydrocarbons. B – Temperature dependence of the wavenumber (cm-1) for the asymmetric CH stretching vibrations (CHa band) in the cell wall material from dormant spores.

Supplementary Table S1. Sequences of the used primers

Primer name	Sequence 5′ – 3′	Description
Inverse primer forward 1	GAATTCTCAACATAGGGTGAAACAATGAC	1st fw primer. Anneals to the end of the gene. The last 4 nucleotides overlap with the second fw primer (in red).
Inverse primer forward 2	TGACAGTCGAATGCTATATC	2nd fw primer. Anneals more to the beginning than the previous primer.
Inverse primer reverse 1	GAATTCGTTGATTTGATAGTTGGCTTTGG	1st rv primer. Anneals to the beginning of the gene. The last 6 nucleotides overlap with the second rv primer (in red).
Inverse primer reverse 2	CTTTGGAAAGCCTGCTTGTG	2nd rv primer. Anneals more to the end than the previous primer.
Disruption forward primer 1	AAGCTTGGAACGTGTACTGATGG	1st fw primer. Contains the HindIII site at the 5′ end (in blue).
Disruption forward primer 2	GCTAGCTCACTACAAACTCCC	2nd fw primer. Contains the NheI site at the 5′ end (in blue).
Disruption reverse primer 1	TCTAGACCTATTGAAGATCCCTTC	1st rv primer. Contains the XbaI site at the 5′ end (in blue).
Disruption reverse primer 2	AGATCTAGCATTCGACTGTCATTG	2nd RV primer. Contains the BglII site at the 5′ end (in blue).
M13/pUC sequencing primer (-20) (17-mer) (Universal)	CTAAAACGACGGCCAGT	Primer used for sequencing Binds at the end of Puc20 (Biolabs).
M13/pUC reverse sequencing Primer (-48) (24-mer) (RACE)	AGCGGTAACAATTTCACACAGCA	Primer used for sequencing Binds at the beginning of Puc20 (Biolabs).
**Splinkerettes**		
Splinktop	CGAATCGTAACCGTTCGTACGAGAATTCGTAC GAGAATCGCTGTCCTCTCCAACGAGCAAGG	Starts with in blueprimer n1 and in red primer n2. The end is complemtary with the bottom.
Splinkbottom	CTAGCCTTGGCTCGTTTTTTTTTGCAAAAA	Starts with the restriction site of BGlII and forms a hairpin structure at the end
Splinkerette primer n1	CGAATCGTAACCGTTCGTACGAGAA	Primer used for the first PCR. Anneals to Splinktop
Splinkerette primer n2	TCGTACGAGAATCGCTGTCCTCTCC	Primer used for the second PCR. Anneals to Splinktop

Supplementary Table S2. Appearance of *Talaromyces macrosporus* cultures on many types of growth medium after various incubation times.

Sample	Medium	Time (h)	Appearance
**1**	Malt extract	48	Only hyphae
**2**	Malt extract	192	Formation of ascomata
**3**	Cherry medium	192	Formation of firm hyphal mat ;fruit-body formation cannot be excluded; strong pigment formation
**4**	Horse dung medium	192	Many conidiophores; low biomass; sometimes clustering of hyphae
**5**	Hay extract	192	Distinct formation of ascomata
**6**	Diluted malt pepton	117	Low biomass; aerial hyphae and conidiophores
**7**	Malt extract	65	Hyphal mat; no fruiting bodies visible
**8**	Acidified malt extract	117	Aerial mycelium; less formation of fruit bodies compared to malt extract; pigment formation
**9**	Horse dung medium	65	Low biomass mycelium; only conidiophores
**10**	Horse dung medium	117	Low biomass mycelium; only conidiophores
**11**	Hay extract	117	Ascomata formation starts; also conidiophore formation
**12**	Cherry medium	117	Hyphal mat without ascomata; presence of conidiophores; strong pigment formation.
**13**	Diluted malt pepton	65	Hyphal mat
**14**	Hay extract	65	Hyphal mat
**15**	Horse dung medium	68	Hyphal mat with conidiophores

**16**	Oatmeal medium	71	Hyphal mat; aerial hyphae
**17a**	Oatmeal medium	116	Start of ascomata formation
**17b**	Oatmeal medium	116	Start of ascomata formation
**18**	Oatmeal medium	165	Formation of ascomata; some pigment formation
**19**	Oatmeal medium	240	Formation of ascomata; some pigment formation
**20a**	Oatmeal medium	408	Mature ascomata; ascospores with ornamentation
**20b**	Oatmeal medium	408	Mature ascomata; ascospores with ornamentation

Supplementary Table 3. Parameters of the logistic fitting of FTIR data

Spores	Band	A1	A2	x0	p	EC20	EC 80
Dormant	CH asymm	2918.487	2924.227	79.9	5.06	60.7	105.1
Activated	CH asymm	2919.206	2924.325	82.6	5.01	62.6	108.9

Reference:

Wolkers, W.F. , Hoekstra, F.A., 1995. Aging of dry desiccation tolerant pollen does not affect protein secondary structure. *Plant Physiol.* **1995**, *109*, 907–15.
